# ‘My people perish for lack of knowledge’: barriers and facilitators to integrated HIV and hypertension screening at the Kenyatta National Hospital, Nairobi, Kenya

**DOI:** 10.1136/openhrt-2022-002195

**Published:** 2023-01-26

**Authors:** Beatrice Wamuti, Mercy Owuor, Christine Magambo, Margaret Ndegwa, Betsy Sambai, Tecla M Temu, Carey Farquhar, David Bukusi

**Affiliations:** 1 Department of Global Health and Population, Harvard University T H Chan School of Public Health, Boston, Massachusetts, USA; 2 Independent Qualitative Researcher, Nairobi, Kenya; 3 Voluntary counselling and testing (VCT) and HIV prevention unit, Kenyatta National Hospital, Nairobi, Kenya; 4 University of Washington - Kenya, Nairobi, Kenya; 5 Department of Global Health, University of Washington, Seattle, Washington, USA; 6 Department of Medicine, University of Washington, Seattle, Washington, USA; 7 Department of Epidemiology, University of Washington, Seattle, Washington, USA

**Keywords:** public health, delivery of health care, quality of health care

## Abstract

**Introduction:**

HIV and cardiovascular disease (CVD) are the two main causes of death in Kenya with hypertension as CVD’s leading risk factor and HIV infection a risk factor for hypertension. We qualitatively evaluated the feasibility of integrated HIV and hypertension screening at Kenyatta National Hospital.

**Methods:**

We conducted two focus group discussions (FGDs) in November 2020 (female FGD: n=7; male FGD: n=8) to elicit facilitators, barriers and viability of integrated diagnosis and management of both conditions at HIV testing service (HTS) facilities. Participants were selected using convenience sampling and were not pair matched. All participants had received HTS. All female clients had confirmed hypertension, while male relatives had been contacted for HIV and hypertension screening through a modified assisted partner services model—where a trained healthcare provider supports notification. Transcripts were coded independently, and the codebook was developed and revised through consensus discussion. Data were analysed using thematic content analysis.

**Results:**

Main barriers to diagnosis and management included limited public awareness of hypertension risk factors and on improved treatment outcomes for those on lifelong HIV treatment, high cost of hypertension care despite free HIV care and healthcare system challenges especially medication stockouts. Strong support systems at family and healthcare levels facilitated care and treatment for both conditions. Participants recommended improved public awareness through individual-level communication and mass media campaigns, decentralised screening services for both HIV and hypertension, and either free or subsidised hypertension care services delivered alongside HIV treatment services. Most felt that an integrated HIV and hypertension service model was viable and would improve healthcare outcomes.

**Conclusion:**

Patient-centred care models combining HIV and hypertension services hold promise for integrated service delivery.

**WHAT IS ALREADY KNOWN ON THIS TOPIC**

- Hypertension is the leading risk factor for cardiovascular disease globally and in Kenya.
- There is low awareness of hypertension status despite high levels of HIV awareness in the country.

**WHAT THIS STUDY ADDS**

- Little investment had been made towards supporting hypertension screening compared with the resources allocated towards HIV screening.
- Improving public-level awareness, increasing the availability of screening services, and integrating care and treatment for both conditions would go a long way in improving outcomes for patients and their families.

**HOW THIS STUDY MIGHT AFFECT RESEARCH, PRACTICE OR POLICY**

- Integration of hypertension and HIV screening in low-income and middle-income countries offers an opportunity to leverage well-established HIV infrastructure.
- Qualitative research incorporating healthcare worker and policy maker perspectives on integrated HIV and hypertension screening, and on evaluating integration of other non-communicable diseases (for example diabetes mellitus, cancer) to HIV is required.

## Introduction

Cardiovascular disease (CVD) is the leading cause of death worldwide with more than 75% of these deaths occurring in low-income and middle-income countries.^1^ Hypertension is the leading associated risk factor for CVD globally and it is most common underlying risk factor in sub-Saharan Africa (SSA). In Kenya, approximately 25% of adults are hypertensive, with 15% of those with hypertension aware and only 27% of those aware on treatment with about half of them achieving blood pressure (BP) control.^2 3^ Few participants are aware of the risk factors for developing hypertension. Studies indicate that men are less likely to be aware of their hypertensive status than women (9% vs 23%)^2^ with many individuals in the region citing stress and old age as the primary causes for hypertension.^4^

HIV prevalence in Kenya among adults aged 15 years and older is about 5% with approximately 1.5 million individuals infected, 80% aware of their status, 96% of those aware on treatment and 91% of those on treatment virally suppressed.^5^ HIV infection is known to be a risk factor to the development of hypertension and other CVDs in persons living with HIV (PLWH) on combined-antiretroviral therapy (ART) and those who are treatment-naïve through derangement of lipid metabolism, immune activation and inflammation.^6^ While overall awareness of HIV status is higher than that for hypertension, gender differences in HIV awareness persist with men less likely to know their HIV status than women (73% vs 83%).^5^ Due to higher awareness among PLWH compared with hypertensive individuals, existing HIV infrastructure can be leveraged to improve hypertension screening. Therefore, targeting hypertensive patients within HIV testing service (HTS) settings can potentially increase screening and promote early diagnosis and management of hypertension among individuals with or at risk for HIV infection.

One of the strategies used to increase HIV awareness among individuals at risk for HIV infection is HIV assisted partner services (aPS). aPS is defined as a process in which consenting HIV-positive individuals are assisted by a trained healthcare provider to disclose their status or to anonymously notify their sexual and/or drug injecting partner(s) of their potential exposure to HIV.^7^ Several studies in SSA have shown that aPS is an effective, safe and cost-effective intervention to increase HIV testing among individuals unaware of their status, and increase linkage to care among diagnosed PLWH not on treatment.^8–10^

We hypothesised that the aPS model can potentially provide an efficient, non-threatening means to reach individuals for concomitant HIV testing and hypertension screening. We conducted a qualitative analysis to evaluate the feasibility of integrated HIV and hypertension screening in a modified aPS project at the Kenyatta National Hospital (KNH) Voluntary Counselling and Testing Centre (VCT).

## Methods

### Study site and design

This qualitative analysis was conducted in November 2020 within the modified aPS study at the KNH VCT, Nairobi, Kenya. KNH was selected since it is the largest public healthcare facility in the country with a diverse population of clients with varying demographic characteristics.^11^ In the modified aPS study, we recruited women with hypertension receiving HTS (female index clients) who may or may not have been HIV-positive. We obtained contact information of their close male relatives including husbands due to associated genetic and lifestyle risk factors for hypertension. Male relatives were traced, notified, counselled and screened for both HIV and hypertension. All participants were at least 35 years of age. We recruited non-pregnant female index clients to avoid the risk of pregnancy-related hypertension. Enrolled participants were followed up at 3 months to assess dietary and lifestyle management, HIV management for those who were HIV-positive and BP control for those who had were hypertensive.

### Study participants

Using convenience sampling, female index clients and male relatives were selected to participate in two in-person focus group discussions (FGDs). FGDs were separated by gender to understand barriers and facilitators to hypertension screening within HIV testing facilities. FGDs were preferred for their wider insights on both conditions allowing participants build on each other’s comments.^12^ Male and female participants were interviewed separately to allow unhindered discussion of sociocultural beliefs surrounding both conditions and were not pair matched to allow varied views from different family dynamics. Convenience sampling was used due to logistical challenges of obtaining participants with COVID-19 social distancing restrictions at the time of the study.^13 14^

### Study procedures and data collection

Enrolled participants were approached to participate in the FGDs lasting approximately 2 hours. The FGDs were conducted in either English or Kiswahili by a qualified female qualitative researcher with a Master of Science degree in community health and development (MO). To ensure consistency and completeness, a semistructured FGD guide was used to direct the interviews. After obtaining informed consent from the participants, the FGDs were audiorecorded, transcribed by a transcriber familiar with the two languages, and verified to eliminate any inconsistencies.

### Data analysis

Recorded interviews and transcripts were assigned identification numbers and personal identifying information removed. Transcripts were then coded independently by BW and MO using inductive coding. Emerging themes were used to develop the codebook, which was then revised through consensus discussion. Interview transcripts were analysed using inductive thematic analysis by using ATLAS.Ti V.8.4.4 and Microsoft Excel. Participant characteristics and selected interview quotes were presented using tables and quotes.

## Results

### Sociodemographic characteristics

Fifteen individuals participated in the two FGDs, seven female index clients and eight male relatives (table 1). Mean age for the female index clients was 51 years, all were hypertensive, one was HIV-positive, most were either single or in married–monogamous relationships and most had not completed secondary education. Mean age among the eight male relatives was 49 years, all were HIV negative and in married monogamous relationships, half had hypertension and most had completed secondary education.

**Table 1:** Participant characteristics

Variable	Description	Female (7)	Male (8)
Age	Mean age, years	51 years	49 years
Hypertension status	Hypertensive	7	4
	Not hypertensive	–	4
HIV status	Positive	1	–
	Negative	6	8
Marital status	Single	2	–
	Married–monogamous	2	8
	Divorced	1	–
	Widow	1	–
	Married polygamous	1	–
Education	Some primary school but did not complete	1	–
	Completed primary education	3	1
	Some secondary but did not complete	2	1
	Completed secondary education	–	5
	Postsecondary education	1	1

### Overall themes

**Figure 1:**
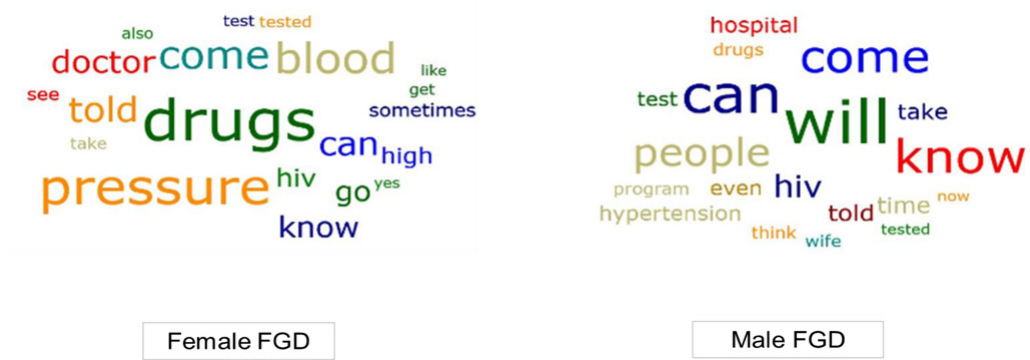
Word clouds comparing key themes from the male and female FGDs. FGDs, focus group discussions. *Females had major concerns around drugs (hypertension medication), high blood pressure, and seeing the doctor at the clinic. Men were more concerned about their agency (will), knowledge (know), and ability (can) to attend (come) clinic visits. Since all female index clients were hypertensive, they were more concerned with care provision, while male relatives seemed eager to take charge of their health through informed decision making.*

The main themes identified from the FGDs are summarised in table 2. Since all female index clients were hypertensive, they focused more on service delivery especially medication supply (figure 1). Male relatives discussed their awareness of hypertension and how they dealt with it as individuals and in their families.

**Table 2:** Key themes

Themes	Subthemes
Barriers	Limited public awareness on hypertension risk factors and on improved treatment outcomes for those on lifelong HIV treatment. High cost of hypertension care compared with free HIV care. Healthcare system challenges especially medication stockouts for hypertension.
Facilitators	Strong family support for both conditions. Patient-centred healthcare system.
Recommendations	Improve public awareness through individual-level communication and mass media campaigns. Decentralise screening services for both HIV and hypertension. Free or subsidised hypertension care services delivered alongside HIV treatment services.

### Barriers to integration

Major barriers to screening, linkage and retention to care for both hypertension and HIV included limited public awareness of key risk factors for hypertension, ongoing HIV-related stigma and fear of death despite the availability of life-prolonging ART, high cost of hypertension care including high consultation and medication fees despite availability of free HIV care and treatment services, and health system challenges especially with the supply of antihypertensive medications.

Prior to participating in the study, most participants were not aware of the risk factors, signs and symptoms of the hypertension, and did not perceive themselves to be at risk. Many especially the younger hypertensive participants were very surprised of their diagnosis as they perceived the condition was related either to old age or stress.

P: I used to think that (hypertension) was for the old people or the rich I did not know that it is for people like us! I: What about now, do you still think that it (hypertension) is a disease for the old people? P: (Laughing) No! Nowadays when someone is sick, I tell them to go and test for hypertension first because it is very common even in young people. - Female FGD

There was also limited awareness of the positive impact of life prolonging ART. Participants associated HIV with death and did not want to have a screening test due to this possibility. Coupled with the fear of death, many also felt that screening for HIV or having a HIV-positive result would lead to HIV-associated stigma at the family and societal level. At the family level, most felt that a HIV-positive result would indicate infidelity on the partner suggesting HIV testing, eventually leading to marital break down, separation and divorce. At the societal level, participants felt that the community would view them as promiscuous. To avoid these fears, they opted out of HIV testing.

You see if I tell him (my husband) that I am (HIV-)positive then that marriage is finished. Even his family members will say that I brought the disease. They will not even think that their son could also bring the disease. - Female FGD

Relative to HIV care and treatment, participants noted that the high cost of hypertensive care and treatment was a major barrier to retention to care. Expenses related to physician review, diagnostic tests and medications made it challenging to adhere to treatment which was exacerbated by job losses due to the COVID-19 pandemic. This contrasted drastically with HIV care. Some participants felt that it was easier for HIV-positive individuals to remain in HIV care due to the free treatment services available through the national HIV programme and the fewer restrictions they had on dietary management, compared with hypertensive clients who incurred fees for service and had tighter dietary restrictions. They felt that this was a potential area for integration where hypertension services could either be subsidised or offered free.

They give HIV patients ARVs (antiretrovirals) for free why can’t they do the same with the hypertension patients? In fact, hypertension is even more serious than HIV. With HIV, you can eat anything but with (high blood) pressure they regulate what you are supposed to eat. So, the government should really consider our situation because even if it is x-ray, the HIV patients do it for free. We are straining so much, and our lives are in danger. Sometimes you come here, and you do not have any money and you are feeling so bad.… So, we are just appealing to the government to consider our case. - Female FGD

Participants on antihypertensive therapy cited frequent medication stockouts at the KNH pharmacy—a government-supported pharmacy where costs are subsidised—as a major issue forcing them to either forgo taking their medications or procure them from private pharmacies at exorbitant prices and questionable quality. Many felt that they had limited options on where to buy high quality affordable medication and would prefer if the government would intervene to ensure continuous supply, similar to the HIV programme.

Sometimes you go to the pharmacy with the prescription, and you are told that “We do not have these drugs here. So, what you need to do is to go and buy them from outside (private pharmacy).” So, when you go and buy them from outside, you find that they are not the same medications as the ones that you have been using. You realize they are counterfeit drugs and they are not working very well for us. - Male FGD

### Facilitators to integration

An overwhelming number of participants in both FGDs cited the presence of a strong support system both at the family and healthcare system level as being crucial facilitators to integrated HIV and hypertension care and treatment. Participants felt that unconditional emotional, psychological, physical, mental and financial support from their partners and family members was crucial in encouraging them to seek care and adhere to treatment, regardless of the illness. Despite societal stereotypes on male roles in the family, for example, being an emotionally distant financial provider with minimal engagement in household duties, male participants without hypertension felt that they were fortunate to be healthy and were more than happy to support their families, knowing that later on they might need the same support in case they too fell ill.

This is teamwork and when she is playing defense, you can play middle field and score. No one needs to know what is going on in your house or that you are washing dishes. So as men we should give the support that is deserved because at the end of the day if you do good it comes back to you. … Don’t take it that she is weak today and frustrate her. You will need her tomorrow when you are weak as well. - Male FGD

Participants felt that a patient-centred healthcare system was crucial in facilitating integrated diagnosis, linkage and retention to care. Through the modified aPS model, the healthcare providers not only educated participants on both conditions, but also offered screening, referral, lifestyle management and treatment adherence services. Participants saw this patient-centred approach as novel and pivotal for a healthcare system that offers curated information for their unique circumstances.

The doctor always calls to ask how I am doing and what I am supposed to do or even if I need to do some exercise. That is one way of bringing us closer to the health personnel. It makes you feel you are important in this life. It also makes you know that at least if you have a problem then there is someone to turn to for solutions. That is one major benefit that I have seen. - Female FGD

### Recommendations

Participants felt that public education on both HIV and hypertension was needed at the individual level—during interactions with healthcare providers, and at the community level—through mass media campaigns. Most were interested in information on risk factors, signs and symptoms, and management of hypertension. On HIV, many participants felt that the focus had shifted towards the COVID-19 pandemic and away from mass media HIV awareness campaigns that had been the norm. This information gap had led to riskier sexual behaviour due to misconceptions that HIV was no longer a public health problem.

… I can say that even the Bible says that “my people perish because of lack of knowledge.” If we get this information and even if it doesn’t help us, we can use the information to help others. All of us here did not have the knowledge before, but because we have been attending clinics and we are taught and have been going to some seminars here and there, you find we are knowledgeable about these things. - Male FGD

Participants recommended mandatory HIV and hypertension screening at every encounter with the healthcare system, whether at the facility or community level. Many did not view it as coercion to have mandatory testing for both conditions. Instead, they saw it as a missed opportunity not being screened whenever they encountered the healthcare system that led to tragic and expensive consequences for example, renal failure, strokes and heart attacks. They also felt that county-level facilities should be adequately equipped with BP machines, HIV test kits and antihypertensive medications to support HIV and hypertension screening and treatment at the community level so that participants did not have to incur costs travelling to higher level facilities seeking diagnosis and treatment.

I think it is not an issue of agreeing or not. You know when a policy or rule is put in place then you just know that “If I go for treatment in the hospital then I must be tested for this and that.” There is no option on that. [Interviewer]: Won’t they feel that they are being forced? [Chorus]: No, it is not forcing. [Participant]: Now why should you beat around the bush only to come here to Kenyatta (National Hospital) to be tested at last? - Male FGD

HIV care was offered free of charge while hypertensive patients incurred costs for hypertension care and treatment. Participants recommended subsidies to offset or totally eliminate the costs of consultations, medications and diagnostic tests. Most had subscribed to the National Hospital Insurance Fund (NHIF)—Kenya’s national healthcare insurance programme—only to find out that it did not cover outpatient visits, which they saw as a missed opportunity in addressing this double burden of disease.

You see it is pointless to pay for the insurance (NHIF) which you are not using because sometimes you can even take five years without falling sick (in-patient cost) but then you eventually come here for treatment, you have to pay seven hundred shillings every month (~$7 outpatient cost) when you are attending clinics. - Male FGD

### Viability of a modified aPS model to support HIV and hypertension screening

Majority of the participants appreciated receiving HIV and hypertension screening through the modified aPS model. Most of them felt that the existing healthcare system did not offer personalised healthcare support and so having a dedicated healthcare provider for ongoing counselling on lifestyle management and treatment adherence was a major advantage of the programme.

The doctor always calls to ask how I am doing and what I am supposed to do or even if I need to do some exercise. That is one way of bringing us closer to the health personnel. It makes you feel you are important in this life. It also makes you know that at least if you have a problem then there is someone to turn to for solutions. That is one major benefit that I have seen. - Female FGD

## Discussion

This study provides client perspectives on an integrated HIV and hypertension screening model using aPS. Most participants felt that an integrated HIV and hypertension service model was viable and would greatly improve healthcare outcomes. Strong support systems at family and healthcare levels facilitated care and treatment for both conditions. However, key barriers to integration included limited public awareness of hypertension risk factors and treatment outcomes for those on lifelong HIV treatment, high cost of hypertension care despite free HIV care and healthcare system challenges, especially medication stockouts. Participants recommended improved public awareness through individual-level communication and mass media campaigns, decentralised screening services for both HIV and hypertension, and either free or subsidised hypertension care services delivered alongside HIV treatment services.

Strong support systems at the family and healthcare system levels were major facilitators to integrated care and management. Contrary to popular belief on separate gender roles at the family level, majority of the male FGD participants were comfortable supporting their spouses in household chores. Many felt that societal dogma against men contributing to domestic duties were outdated, and most were more than willing to support spousal care and treatment. While different integration formats are yet to be evaluated in SSA, participants in Tanzania felt that a peer support system supporting at-risk group members to change risky behaviours and maintain healthy lifestyle behaviours would help improve care and treatment, similar to feedback from our FGDs.^15^ At the healthcare system level, participants advocated for the modified aPS model as it offered a patient-centred approach to managing HIV and hypertension.^16 17^ The availability of a healthcare personnel familiar with the patient’s case was seen as a major advantage, and participants felt that it was easier to receive ongoing support for care retention.

Participants reported low awareness of the risk factors, signs and management of hypertension similar to other studies conducted in the region^2–4 18^ despite large public awareness on HIV. The existing HIV infrastructure can support increased public education for hypertension and other non-communicable diseases (NCDs) at the patient, family, community and healthcare system levels, for example, through interpersonal communication between the patient and provider, community screening engagements, mass media campaigns and training of healthcare providers on screening.^16^ Such platforms can potentially support screening for other NCDs, for example, diabetes mellitus and cancer.^3^ Surprisingly, participants were supportive of mandatory opt-out HIV and hypertension screening at each encounter with the healthcare system preferring it to the missed opportunities for early diagnosis and treatment of both conditions that have tragic long-term health and financial consequences. Policy makers may also consider instituting opt-out screening measures to promote early diagnosis and treatment, but this must consider three of the five Cs of HIV testing, that is, consent, counselling and confidentiality, to ensure clients are fully aware of the various care options should they screen positive.^19^

Despite high HIV awareness, stigma and fear of death persisted especially due to the decline of HIV awareness campaigns during the COVID-19 pandemic indicating the need to revamp and sustain mass education efforts. This ongoing stigma is similar to other SSA settings where PLWH saw the intersection between HIV and NCDs as a challenge due to the additional pill burden, clinician visits and mental distress.^20^ Such individuals will require multifaceted strategies to address structural and societal barriers to integrated HIV and hypertension management, for example, expedited clinic reviews, counselling support and multidisciplinary management at the individual level, clear implementation strategy with standardised care checklists and service coordinators at the healthcare system level, and proactive public awareness campaigns at the community level.^16 17 21^

High costs of hypertension care and treatment and supply chain challenges were major barriers to ongoing hypertension management. These stood against a backdrop of free HIV care and treatment services where service interruptions were infrequent. Participants proposed holistic healthcare system improvements to offset costs of hypertension services and promote patient-centred care models, for example, free or subsidised NCD care through the NHIF. Similar to a Tanzania study evaluating hypertension care for PLWH, providers noted system-related capacity limitations (staff, medications) and high costs of treatment as the underlying individual-level barriers and recommended prioritisation of resources and funding towards hypertension care.^18^ Ongoing NHIF reforms to expand care for NCDs signal hope to patients,^22^ and as policy makers consider integrated NCD management within and outside the existing HIV infrastructure, subsidies towards clinician consultation, diagnostics and medication will go a long way in improving the care cascade.

There were several strengths to this study. First, we evaluated an integrated HIV and hypertension aPS model contributing to literature on its feasibility in Kenya. Second, we conducted the study at KNH, the largest teaching and referral hospital in Kenya, to get a representative sample of the population and improve generalisability of study results. Third, we conducted the male and female FGDs separately to reduce potential for social desirability bias and obtain a better sense of societal norms and their influence on screening, care and treatment for both conditions. Among the limitations, we did not include provider or healthcare system-level perspectives due to budgetary constraints. These views would have given broader perspectives of integrating NCDs to existing HIV infrastructure. Also, by conducting FGDs, we were not able to get individual-level perspectives from interviewees. However, the findings from our study provide insight to suitable formats for integrated service delivery.

## Conclusion

Low public awareness on hypertension and HIV-associated stigma persists in Kenya. Integrating services into the existing HIV infrastructure will go a long way in promoting population-level screening, diagnosis, linkage and management not only for hypertension but also other NCDs.

## Data Availability

Data are available on reasonable request.
